# Impact of the COVID-19 outbreak on acute stroke care

**DOI:** 10.1007/s00415-020-10069-1

**Published:** 2020-07-20

**Authors:** L. A. Rinkel, J. C. M. Prick, R. E. R. Slot, N. M. A. Sombroek, J. Burggraaff, A. E. Groot, B. J. Emmer, Y. B. W. E. M. Roos, M. C. Brouwer, R. M. van den Berg-Vos, C. B. L. M. Majoie, L. F. M. Beenen, D. van de Beek, M. C. Visser, S. M. van Schaik, J. M. Coutinho

**Affiliations:** 1 Department of Neurology, Amsterdam UMC, University of Amsterdam, Amsterdam Neuroscience, Amsterdam, The Netherlands; 2 Department of Neurology, OLVG, Amsterdam, The Netherlands; 3 Department of Neurology, Amsterdam UMC, Vrije Universiteit Amsterdam, Amsterdam Neuroscience, Amsterdam, The Netherlands; 4 Department of Radiology and Nuclear Medicine, Amsterdam UMC, University of Amsterdam, Amsterdam, The Netherlands

**Keywords:** Stroke, Acute care, Quality, Reperfusion

## Abstract

**Background and purpose:**

There are concerns that the coronavirus disease 2019 (COVID-19) outbreak negatively affects the quality of care for acute cardiovascular conditions. We assessed the impact of the COVID-19 outbreak on trends in hospital admissions and workflow parameters of acute stroke care in Amsterdam, The Netherlands.

**Methods:**

We used data from the three hospitals that provide acute stroke care for the Amsterdam region. We compared two 7-week periods: one during the peak of the COVID-19 outbreak (March 16th–May 3th 2020) and one prior to the outbreak (October 21st–December 8th 2019). We included consecutive patients who presented to the emergency departments with a suspected stroke and assessed the change in number of patients as an incidence-rate ratio (IRR) using a Poisson regression analysis. Other outcomes were the IRR for stroke subtypes, change in use of reperfusion therapy, treatment times, and in-hospital complications.

**Results:**

During the COVID-19 period, 309 patients presented with a suspected stroke compared to 407 patients in the pre-COVID-19 period (IRR 0.76 95%CI 0.65–0.88). The proportion of men was higher during the COVID-19 period (59% vs. 47%, *p* < 0.001). There was no change in the proportion of stroke patients treated with intravenous thrombolysis (28% vs. 30%, *p* = 0.58) or endovascular thrombectomy (11% vs 12%, *p* = 0.82) or associated treatment times. Seven patients (all ischemic strokes) were diagnosed with COVID-19.

**Conclusion:**

We observed a 24% decrease in suspected stroke presentations during the COVID-19 outbreak, but no evidence for a decrease in quality of acute stroke care.

## Introduction

The coronavirus disease 2019 (COVID-19) outbreak has put health care systems worldwide under enormous pressure, potentially impairing the quality of care for patients with acute cardiovascular conditions [1–4]. At the same time, studies suggest that COVID-19 may increase the risk of thromboembolic diseases, including stroke [5, 6]. Intravenous thrombolysis (IVT) and endovascular thrombectomy (EVT) are the cornerstone of acute ischemic stroke treatment, but their effects on clinical recovery are highly time-dependent [7, 8]. We assessed the impact of the COVID-19 outbreak on trends in hospital admissions for (suspected) stroke, patient characteristics, and workflow parameters of acute stroke care in Amsterdam, the Netherlands.

## Methods

We conducted a retrospective multicenter cohort study, using data from the prospective stroke registries of the only three hospitals (2 primary, 1 comprehensive stroke center) that provide acute stroke care for the Amsterdam area (approximately 1.1 million inhabitants). We included consecutive patients who were presented to the emergency departments of these hospitals with acute-onset focal neurological symptoms suggestive of an acute stroke (= suspected stroke or code stroke). In-hospital cases were also included. To verify completeness of the stroke registries, we cross-referenced the data with all suspected stroke pre-notifications during the two time periods. We compared data of two time periods of 7 weeks: one during the peak of the COVID-19 outbreak in The Netherlands (March 16th–May 3th 2020) and one prior to the worldwide outbreak of the disease (October 21st–December 8th 2019, pre-COVID-19/control period). March 16th was the first day that strict nationwide lockdown measures were implemented, including working from home and closure of schools and restaurants. The sample size of two 7-week periods was based on an estimated 25% decrease in the weekly number of suspected stroke presentations during the COVID-19 outbreak (from 64 to 48 presentations per week, alpha 0.05, and power of 0.80).

Diagnoses were categorized as: ischemic stroke, transient ischemic attack (TIA), intracranial hemorrhage (intracerebral or subarachnoid hemorrhage), or others (e.g., seizure, functional neurological symptoms, and peripheral vestibular disorder). Patients who were clinically suspected of having COVID-19 were tested using PCR. Patients with PCR confirmed COVID-19 were admitted to designated COVID-19 wards. The study was approved by the ethical review board of each hospital and the need for written informed consent was waived. Study outcomes were: (1) change in the number of emergency department presentations; (2) change in proportion of stroke patients treated with IVT and EVT; (3) change in IVT and EVT treatment times; and (4) in-hospital complications.

Incidence-rate ratios (IRR) comparing the COVID-19 period to the pre-COVID-19 period were calculated using a Poisson regression. For the other outcomes, we performed independent samples *t* test, Mann–Whitney *U* test, Fishers’ exact test, or Chi-square test, as appropriate. Statistical analyses were done with R software (Version 3.6.1, R Foundation).

## Results

In total, 309 patients presented with a suspected stroke during in the COVID-19 period compared to 407 during the pre-COVID-19 control period [IRR 0.76, 95% confidence interval (CI) 0.65–0.88, Table 1]. During the COVID-19 period, 212 patients were diagnosed with an ischemic stroke or TIA compared to 248 in the control period (IRR 0.85, 95%CI 0.71–1.02), and 20 patients compared to 42 were diagnosed with an intracranial hemorrhage (IRR 0.48, 95%CI 0.28–0.81). Baseline characteristics were mostly similar, but patients in the COVID-19 cohort were more often men (59% vs. 47%, *p* < 0.001, Table 2). There was no difference in NIHSS score (4 vs. 4, *p* = 0.55), proportion of large vessel occlusions (26% vs. 22%, *p* = 0.44), or onset-to-door time (187 vs. 150 min, *p* = 0.39). In the COVID-19 cohort, a total of 60/309 (19%) patients were clinically suspected of having COVID-19 and were tested by means of PCR. Of these, COVID-19 was confirmed in 7 patients (2%, three men). All seven patients had an ischemic stroke and two had a large vessel occlusion. One patient was treated with endovascular treatment and two with intravenous thrombolysis. One patient was admitted to a COVID-19 designated intensive-care unit and died during admission. None of the other patients with PCR confirmed COVID-19 died during admission or within 7 days.

**Table 1:** Incidence-rate ratios of admissions per 7 weeks for suspected stroke and final diagnoses during the COVID-19 pandemic as compared with the pre-COVID-19 period

	COVID-19 cohort (*n* = 309)	Pre-COVID-19 cohort (*n* = 407)	Incidence rate ratio (95%CI)
All suspected stroke presentations, *n* (%)	309	407	0.76 (0.65–0.88)
Ischemic stroke or TIA, *n* (%)^a^	212/309 (69)	248/407 (61)	0.85 (0.71–1.02)
Intracranial hemorrhage, *n* (%)	20/309 (6)	42/407 (10)	0.48 (0.28–0.81)
Other, *n* (%)^b^	77/309 (25)	115/407 (28)	0.66 (0.50–0.89)

^a^Number of ischemic strokes and TIAs were (COVID-19 vs. pre-COVID-19): ischemic stroke (180 vs. 194), and TIA (32 vs 54)

^b^Main other diagnoses (COVID-19 vs pre-COVID-19): seizure (13 vs. 16), peripheral vestibular syndrome (11 vs. 15), benign headache syndromes (4 vs. 5), functional neurological symptoms (11 vs. 15), and cerebral venous thrombosis (0 vs. 2)

**Table 2:** Baseline characteristics

	COVID-19 cohort (*n* = 309)	Pre-COVID-19 cohort (*n* = 407)	*p* value
Mean age in years (± SD)	70 (14)	69 (16)	0.18
Male, *n* (%)	183/309 (59)	190/407 (47)	** < 0.001**
Mean systolic blood pressure (mmHg ± SD)^a^	154 (28)	155 (28)	0.31
Mean diastolic blood pressure (mmHg ± SD)^b^	90 (18)	87 (19)	0.09
Median NIHSS (IQR)^c^	4 (2–9)	4 (2–7)	0.55
Median O_2_ saturation (IQR)^d^	98 (96–99)	97 (96–99)	0.57
Fever on admission (> 38 degrees Celsius, *n* (%))	5/226 (2)	12/289 (4)	0.22
Medical history			
Stroke or TIA, *n* (%)	101/309 (33)	132/407 (32)	0.94
Atrial fibrillation, *n* (%)	55/309 (18)	56/407 (14)	0.15
Diabetes mellitus, *n* (%)	58/309 (18)	66/407 (16)	0.43
Hypertension, *n* (%)	153/309 (50)	178/407 (44)	0.12
Hypercholesterolemia	81/309 (26)	76/407 (19)	**0.02**
Coronary artery disease	53/309 (17)	54/407 (13)	0.15
Smoking, *n* (%)	92/309 (29)	123/407 (30)	0.90
Medication use			
Statin, *n* (%)	113/309 (37)	155/407 (38)	0.67
Anticoagulation, *n* (%)	31/309 (10)	52/407 (13)	0.26
Antiplatelet, *n* (%)	95/309 (31)	117/407 (28)	0.56
Anti-hypertensive, *n* (%)	162/309 (52)	210/407 (52)	0.98
Laboratory on admission			
Mean thrombocyte count × 10^3^ per mm^3^ (± SD)^e^	237 (68)	243 (79)	0.28
Mean leucocyte count × 10^3^ per mm^3^ (± SD)^f^	9.3 (6.6)	9.1 (9.2)	0.77
Mean C-reactive protein in mg/L (± SD)^g^	13.4 (41.4)	10.7 (30.8)	0.24
Mean glucose in mmol/L (± SD)^h^	7.9 (3.4)	7.6 (3.8)	0.35
Onset of symptoms			0.07
Witnessed, *n* (%)	170/309 (56)	242/407 (60)	
Wake-up, *n* (%)	62/309 (20)	55/407 (14)	
Unknown, *n* (%)	73/309 (24)	104/407 (27)	
In-hospital occurrence of ischemic stroke, *n* (%)	10/309 (6)	6/407 (3)	0.36
Process measures			
Onset-to-door time (median, minutes, IQR)^i^	187 (71–606)	150 (75–544)	0.39
Door-to-CT brain (median, minutes, IQR)^j^	13 (8–22)	12 (8–21)	0.42
Large vessel occlusion, *n* (%)	46/180 (26)	42/194 (22)	0.44

Missing values, *n* (%): ^a^36 (5), ^b^36(5), ^c^Ischemic stroke patients only, no missing values. ^d^83 (12), ^e^71 (10),^f^44 (6), ^g^71 (10), ^h^83 (12), ^I^For patients with unwitnessed onset of symptoms, the last-seen-well time was used to calculate the time interval between onset of symptoms and arrival at first hospital, 131 (18), ^j^ 64 (9)

There was no difference in the proportion of stroke patients treated with IVT (28% vs. 30%, *p* = 0.58) or EVT (11% vs. 12%, *p* = 0.82, Table 3) in the COVID-19 and control period, respectively. Treatment times were comparable between periods (door-to-needle time 31 vs. 28 min, *p* = 0.39; first-door-to-groin times 110 min vs. 96 min, *p* = 0.18). Complication rates and discharge destinations also did not differ.

**Table 3:** Other outcomes

	COVID-19 cohort (*n* = 309)	Pre-COVID-19 cohort (*n* = 407)	*p* value
Reperfusion therapy			
IV thrombolysis, *n* (%)	50/180 (28)	59/194 (30)	0.58
Endovascular thrombectomy, *n* (%)	20/180 (11)	23/194 (12)	0.82
Successful reperfusion after endovascular thrombectomy, *n* (%)^a^	16/20 (80)	15/22 (68)	0.38
Process measures			
Door-to-needle time (median, minutes, IQR)^b^	31 (21–51)	28 (21–40)	0.39
First door-to-groin time (median, minutes, IQR)^c^	112 (70–155)	96 (61–128)	0.24
Door comprehensive stroke center to groin time (median, minutes, IQR)^d^	60 (48–78)	61 (48–75)	0.88
Complications			
ICU admission, *n* (%)	16/309 (5)	20/407 (5)	0.87
sICH, *n* (%)	0/309	2/407 (1)	0.38
Mortality within 7 days, *n* (%)	17/201 (9)	19/382 (5)	0.10
Discharge destination			0.44
Home, *n* (%)	157/283 (55)	225/397 (56)	
Nursing home, *n* (%)	9/283 (3)	14/397 (4)	
Rehabilitation center, *n* (%)	37/283 (13)	37/397 (9)	
Other hospital, *n* (%)	64/283 (22)	104/397 (26)	
In-hospital death, *n* (%)	16/283 (6)	17/397 (4)	

Data on reperfusion therapy and process measures only regard patients with ischemic strokes

*IV* indicates intravenous; *IQR* interquartile range; *COVID-19* coronavirus disease 2019; *ICU* intensive-care unit; *sICH* symptomatic intracranial hemorrhage

^a^Defined as extended thrombolysis in cerebral infarction score of 2*b*–3. Missing values, *n* (%):^b^7(6), ^c^for transfer patients, the first-door-to-groin time is calculated as the time interval between presentation at the primary stroke center and groin puncture at the comprehensive stroke center, missing values, *n* (%) 4(9) ^d^0(0), ^e^29 (4)

## Discussion

We observed a 24% decrease in the number of patients with a suspected stroke in the hospitals in the Amsterdam area during the height of the COVID-19 outbreak compared to a pre-COVID-19 control period. The proportion of patients who underwent reperfusion therapy did not change during the outbreak, nor did we observe a difference in treatment times, but the study was not powered for these outcomes. Seven ischemic stroke patients (2%) also had COVID-19.

There are several potential explanations for the decreased number of suspected stroke presentations. First, people may have been more reluctant to call emergency services or go to the hospital during the pandemic out of fear of contracting COVID-19. General practitioners also may have had a higher threshold to refer patients during the outbreak. Second, stroke symptoms are often not recognized by patients themselves and the initiative to seek medical help frequently comes from bystanders. Due to the social distancing measures, some strokes may have remained unrecognized, especially in elderly who are more often socially isolated [9]. Third, stroke symptoms may have been overlooked in patients with suspected COVID-19, especially early in the pandemic when the risk of thromboembolic disease in these patients was not well known. Fourth, we cannot exclude the possibility that social distancing measures somehow decrease the risk of stroke, for instance because of improved air quality or because of a decrease in incidence of other transmissible diseases [10, 11].

Another contributing factor may have been general changes in inpatient and outpatient services of hospitals during the COVID-19 outbreak. In the three participating hospitals, virtually, all outpatient visits were suspended during the outbreak, with the exception of acute outpatient referrals. All other non-acute care was done via telephone. However, since stroke patients in our region rarely are presented through outpatient clinics, the influence of suspension of outpatient services on acute stroke care is probably minimal. None of the hospitals had a reduction in capacity for inpatient stroke, i.e., no change in the number of available stroke beds and no restrictions in stroke services during the outbreak. This included performance quality indicators such as swallowing assessment, access to ancillary exams, and in-hospital rehabilitation which should, therefore, should not have differed between the two time periods, although specific data on these individual performance indicators were not available for comparison.

Of note, the proportion of men who presented to the emergency department was approximately 10% higher during the COVID-19 outbreak. This could indicate that the threshold to come to the hospital was somehow higher for women. Another possible explanation for this observation is the sex-related age disparity in stroke, i.e., that women are generally older than men and may, therefore, more frequently have been socially isolated [12]. This age difference could also imply that women more frequently resided in a nursing home at the onset of symptoms and these patients may have been less likely to be referred to the emergency room for treatment. Unfortunately, data on living conditions prior to the stroke were not available. Men with COVID-19 also appear to have a higher risk of a complicated disease course [13], but the sex ratio of stroke patients with confirmed COVID-19 was not skewed in our study.

One of the strengths of our study is that we used data from the only hospitals that provide acute stroke care in the Amsterdam area. Hence, the decrease in patients cannot be explained by a diversion of patients with a suspected stroke to other hospitals in the region. Our study also has several limitations. First, data collection was retrospective, but the number of patients with missing data was low and by cross-referencing data with emergency room stroke pre-notifications, the chance that we missed patients is small. Second, we report regional and not national data and it is unknown whether the COVID-19 outbreak had a similar effect on acute stroke care in other areas of the country. Third, we were unable to reliably report on key performance quality indicators such as swallowing assessment and in-hospital rehabilitation to assess any difference between the two time periods. Fourth, we did not compare incidences with the exact same time period 1 year before. Instead, we used a time period closer to the outbreak, because the proportion of stroke patients who are treated with reperfusion therapy has lately increased in our region after publication of studies that showed efficacy of IVT and EVT in the extended time-window [14, 15]. Also, the data on whether there is a seasonal effect on the epidemiology of stroke are conflicting, but if such an effect exists, the risk appears to be the highest in May, [16] which could indicate that our study underestimated the decrease in stroke presentation during the COVID-19 outbreak. Fifth, we were unable to reliably report on key performance quality indicators such as swallowing assessment and in-hospital rehabilitation to assess any difference between the two time periods. Finally, we were unable to report data on long-term functional outcome in this study, since the 90 day duration of follow-up has not yet elapsed for patients presenting during COVID-19 outbreak. Future studies should address this, ideally using data from (inter)national collaborations, as functional outcome at 90 days is an important factor in assessing the influence of the COVID-19 outbreak on acute stroke care.

In summary, we found a substantial decrease in the number of suspected stroke presentations during the COVID-19 outbreak in the Amsterdam area, but no evidence for a change in quality of acute stroke care.
